# Protective Effects of Interleukin-37 Expression against Acetaminophen-Induced Hepatotoxicity in Mice

**DOI:** 10.1155/2022/6468299

**Published:** 2022-04-04

**Authors:** Zhiwei Xu, Kan Li, Xiuhe Pan, Jun Tan, Yan Li, Mingcai Li

**Affiliations:** ^1^The Affiliated Lihuili Hospital of Ningbo University, Ningbo Medical Center Lihuili Hospital, Ningbo, China; ^2^School of Medicine, Ningbo University, Ningbo, China; ^3^HwaMei Hospital, University of Chinese Academy of Sciences, Ningbo, China

## Abstract

**Aim:**

Interleukin (IL)-37 is a new anti-inflammatory cytokine of the IL-1 family. This study aimed to determine the effects of IL-37 on acetaminophen (APAP)-induced liver injury.

**Materials and Methods:**

IL-37 plasmids were injected into mice via a tail vein hydrodynamics-based gene delivery.

**Results:**

Our results showed that IL-37 pretreatment significantly decreased serum alanine aminotransferase and aspartate aminotransferase levels, hepatic myeloperoxidase activity, and attenuated the histological liver damage. Compared to the APAP group, IL-37 administration decreased Kupffer cells numbers in the liver of APAP-induced hepatotoxicity in mice. Furthermore, IL-37 pretreatment reduced the expression of proinflammatory cytokines including tumor necrosis factor-*α*, IL-6, IL-17, and nuclear factor-*κ*B (NF-*κ*B) in APAP-induced mice.

**Conclusion:**

These results demonstrate that delivery of IL-37 plasmid can ameliorate APAP-induced liver injury by reducing proinflammatory cytokines production and preventing the activation of the NF-*κ*B signaling pathway. IL-37 may be a promising candidate against APAP-induced liver injury.

## 1. Introduction

Liver is the largest digestive gland and the center of the human energy metabolism, which is closely related to human health. Liver diseases, including viral hepatitis, autoimmune hepatitis, and alcoholic liver disease, represent a significant public health problem, as many can develop into liver failure [1]. Acetaminophen (N-acetyl-p-aminophenol or APAP)-induced hepatotoxicity is also the main cause of acute liver failure. APAP may cause abdominal pain, nausea, and even acute liver failure. APAP antidote N-acetylcysteine is commonly used to treat APAP-induced hepatotoxicity. A better understanding of the mechanism underlying the hepatotoxicity is needed for the generation of more effective therapeutic strategies against the disorder.

Interleukin (IL)-37 (formerly IL-1 family member 7, IL-1F7) is identified as a novel anti-inflammatory cytokine of the IL-1 family, which includes IL-1*α*, IL-1*β*, IL-1 receptor antagonist (Ra), IL-18, IL-33, IL-36*α*, IL-36*β*, IL-36*γ*, IL-36Ra, and IL-38 [2]. At present, no mouse homologue has been identified; however, five different isoforms of human IL-37 have been described (IL-37a–e) [3, 4]. It exists in several organs and tissues including the brain, kidney, heart, bone marrow, and testis in specific isoforms. IL-37 is also expressed in peripheral blood mononuclear cells, monocytes, dendritic cells, and epithelial cells, and almost completely inhibits the synthesis of proinflammatory cytokines [5, 6]. Studies have shown that IL-37 is constitutively expressed in tissues from patients with rheumatoid arthritis compared to health subjects [7]. Moreover, some studies indicate that IL-37 has significant anti-inflammatory effects in models of septic shock, dextran sulfate sodium colitis, ischemia-reperfusion injury, obesity-induced inflammation, allergic airway inflammation, and lung fibrosis [7–15]. These findings tend to imply that IL-37 mediates a negative feedback mechanism to suppress excessive inflammation. It has been reported that IL-37 is protective against hepatic ischemia/reperfusion injury [16], and IL-37 can affect concanavalin A (ConA)-induced hepatitis [17–19]. However, its protective effect on APAP-induced liver injury needs to be elucidated.

In the present study, we found that IL-37 inhibited APAP-induced liver injury in mice. IL-37 treatment exhibited significantly reduced serum alanine aminotransferase (ALT) and aspartate aminotransferase (AST) levels, hepatic myeloperoxidase (MPO) activity, and ameliorated histological liver damage in mice. The protective effect of IL-37 was associated with reduced tumor necrosis factor (TNF)-*α*, IL-6, and IL-17 production.

## 2. Materials and Methods

### 2.1. Reagents and Mice

APAP was purchased from Sigma Chemical Co (St. Louis, MO, USA). Plasmids pcDNA3.1 and pcDNA3.1-IL-37 (6-polyhistidine tag was introduced into the 3′ end of IL-37 cDNA for detection and purification) were preserved in our laboratory. RNAiso Plus reagents were purchased from TaKaRa Bio, Inc (Dalian, China). The HiFiScript 1st strand cDNA synthesis kit was purchased from ComWin Biotech Co., Ltd. (Beijing, China). Radio immunoprecipitation assay (RIPA) lysis buffer was purchased from Beyotime Institute of Biotechnology (Shanghai, China). Bovine serum albumin (BSA) was purchased from Solarbio Science and Technology Co. (Beijing, China).

Six–eight-week-old male BALB/c mice weighing between 18 and 22 g were obtained from the Animal Experimentation Center of Zhejiang Chinese Medical University (Hangzhou, China). Mice were kept with standard laboratory conditions with free access to food and water. The animals were set to adapt to the new environment for seven days before experimental procedures, which was under a protocol approved by the Ethical Committees of Ningbo University School of Medicine.

### 2.2. Recombinant Plasmid Extraction

E. coli TOP10 carrying pcDNA3.1 and pcDNA3.1-IL-37 were respectively large-scale culture, and then plasmids were extracted by EndoFree Maxi Plasmid kit (Tiangen Biotech, Beijing, China). Finally, the plasmids were stored at −80°C for later experiment.

### 2.3. Hydrodynamic Plasmid Injection

Plasmid DNA was introduced into mouse liver using hydrodynamic tail vein injection approach as reported [20, 21]. Briefly, 100 *μ*g/mouse of corresponding plasmid DNA was diluted in 2.0 ml of saline (0.1 ml/g bodyweight) and injected into the mouse via tail vein within a time period of 5–10 s. The mice were typically recovered from the injection within 5–10 min.

### 2.4. Detection of IL-37 mRNA and Protein Expression

Mice were injected with pcDNA3.1-IL-37 plasmids as described above, and then were sacrificed after hydrodynamic procedures at 0, 6, 12, 24, 48, 72, and 96 h. Finally, liver tissues were collected for RNA extraction and the liver homogenates were obtained for enzyme-linked immunosorbent assay (ELISA) at indicated time points. Total RNA from above harvested liver was extracted and the cDNA was synthesized from 4 *μ*g of total RNA using the HiFiScript 1st strand cDNA synthesis kit and amplified by reverse transcription-polymerase chain reaction (RT-PCR). The PCR primers of IL-37 and *β*-actin (Generay Biotech, Shanghai, China) are given in Table 1. Differences in expression were normalized to the *β*-actin signal. The cycles for IL-37 PCR were as follows: 98°C for 5 min, 35 cycles of 98°C for 30 s, 62°C for 30 s, 72°C for 30 s, and then 72°C for 10 min. The conditions for *β*-actin PCR were equal with above, except for annealing temperature of 62.9°C. PCR products were separated by 1.2% agarose gels electrophoresis.

IL-37 concentrations in mouse liver homogenates at 0, 6, 12, 24, 48, 72, and 96 h after hydrodynamic tail vein injection were determined by ELISA kit (CUSABIO, Wuhan, China).

### 2.5. Experimental Design

Mice liver damage was induced by intraperitoneal injection of APAP (400 mg/kg bodyweight) dissolved in pyrogen-free saline as previously described [22, 23]. The animals were euthanized 24 h after APAP administration. Blood and liver were collected for subsequent analysis.

Mice fasted overnight were randomly divided into three groups: pcDNA3.1/saline group, pcDNA3.1/APAP group, and pcDNA3.1-IL-37/APAP group. To assess the protective effect of IL-37 expression on APAP-induced hepatotoxicity, pcDNA3.1-IL-37 plasmid was administrated via hydrodynamic tail vein injection 24 h prior to treatment with APAP as described above. Meanwhile, the pcDNA3.1 empty plasmid was injected according to the same protocol as a control.

### 2.6. Assay for Serum Transaminase Activity

Liver enzymes including ALT and AST activities in serum were determined using ALT and AST reagent kits (Nanjing Jiancheng Bioengineering Institute, Nanjing, China) according to the manufacturer's instructions.

### 2.7. Measurement of Liver MPO Activity

The liver tissues were weighted, and 5% tissue homogenate was prepared; then, the MPO activity in liver samples was measured with commercial kits (Nanjing Jiancheng Bioengineering Institute, Nanjing, China) according to the manufacturer's protocols.

### 2.8. Analysis of Liver Histopathology

Liver tissues were fixed in 10% buffered formalin for 48 h. The tissues were embedded in paraffin and cut at thicknesses of 4 *μ*m. The tissue sections were stained with hematoxylin and eosin (H&E), and histological examination was performed by a pathologist blinded to the experiment.

### 2.9. Immunohistochemistry

For immunohistochemical staining, antigen retrieval was performed by incubating the sections using 10 mM citrate buffer, pH 6 for 15 min at 97°C. Activity of endogenous peroxidase was blocked by incubating sections with 3% v/v hydrogen peroxide for 10 min. Sections were then washed with phosphate buffer solution (PBS) three times and blocked with 5% normal blocking serum at 37°C for 20 min. Next, the sections were incubated overnight at 4°C with primary antibodies including anti-mouse F4/80 (Santa Cruz Biotechnology, Shanghai, China, 1 : 50) or anti-His6 mouse primary antibodies (Beyotime, Beijing, China, 1 : 100). On the second day, sections were washed with PBS and incubated with 1 : 200 diluted HRP-conjugated anti-rat IgG secondary antibodies (Bioss Biotech, Beijing, China) for 1 h at 37°C. Color development was induced using 3, 3′-diaminobenzidine (DAB) substrate (Boster, Wuhan, China) during a 10–30 min incubation period. Using this substrate, specific staining was visualized by light microscopy.

### 2.10. Determination of Cytokine Levels by RT-PCR and ELISA

Total RNA was extracted from the collected liver at 24 h after APAP injection as described earlier. RT-PCR was used to assess the expression levels of inflammatory cytokines in the liver injury. PCR primers (Generay, Shanghai, China) are given in Table 1. PCR products were separated by 1.2% agarose gels electrophoresis. All samples were normalized against the intensity of *β*-actin. Serum cytokine concentrations of TNF-*α*, IL-6, and IL-17 were also determined by ELISA kits (ExCell Bio, Shanghai, China) according to the manufacturer's instructions.

### 2.11. Western Blot

Liver tissues stored at −80°C were minced into small pieces and fully homogenated in 1 ml RIPA lysis buffer with 10 *μ*l phenylmethane-sulfonyl fluoride (PMSF) using a Tissue Tearor (Biospec, Bartlesville, USA). The homogenates were then put on ice for 30 min and centrifuged at 12,000 g at 4°C for 15 min. The concentration of the supernatants was calculated using the bicinchoninic acid (BCA) protein assay (Kaiji, China). The supernatants were then mixed in 4 × loading buffer, boiled for 5 min, and subjected to sodium dodecyl sulfate-polyacrylamide gel electrophoresis (SDS-PAGE). After electrophoresis, proteins were transferred onto polyvinylidene fluoride (PVDF) membranes (Millipore, Bedford, MA, USA). After blocking with 5% BSA in Tris buffered saline with Tween-20 (TBST) (20 mM Tris-HCl, pH 7.5, 500 mM NaCl, and 0.05% Tween-20), membranes were incubated with 1 : 800 diluted nuclear factor-*κ*B (NF-*κ*B), p65, and *β*-actin antibodies (TransGen, Beijing, China) at 4°C overnight, followed with 1 : 5000 diluted horseradish peroxidase (HRP)-conjugated anti-goat IgG secondary antibodies (Bioss Biotech, Beijing, China). Protein bands were visualized by an enhanced chemiluminescence reaction (ECL) (Amersham Pharmacia Biotech, Piscataway, NJ) and analyzed with a Tanon gel image analysis system (Tanon, Shanghai, China).

### 2.12. Statistics

Data were expressed as mean ± SD. Student's *t*-test was used to compare two groups. For multiple comparisons, the one-way ANOVA with Dunnett's method was used. In all analyses, *p* < 0.05 was considered statistically significant.

## 3. Results

### 3.1. IL-37 Expression in Mice by Hydrodynamic Gene Delivery of IL-37 Plasmids

**Figure 1:**
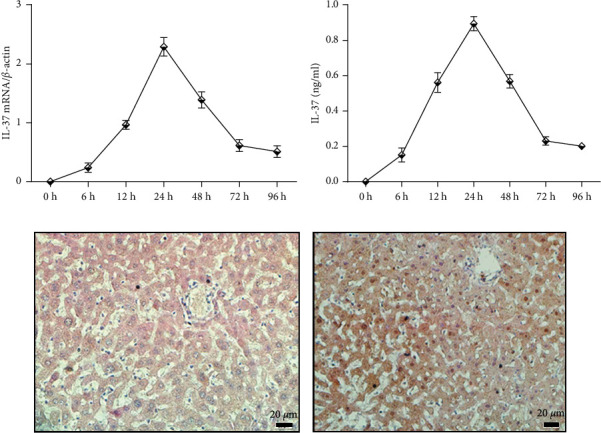
Detection the expression of exogenous IL-37. (a) IL-37 mRNA levels in mouse liver tissues analyzed by RT-PCR at 0, 6, 12, 24, 48, 72, and 96 h after hydrodynamic procedures. Data are expressed as mean ± SD of animals analyzed at each time point (*n* = 5). (b) IL-37 protein levels in liver homogenate analyzed by ELISA at each similar time point. Data are expressed as mean ± SD of animals analyzed at each time point (*n* = 5). IL-37 mRNA and protein levels were normalized with *β*-actin expression. IL-37 protein expression was detected in mouse livers 24 h after injection of empty pcDNA3.1 (c) and pcDNA3.1-IL-37 (d) plasmids using immunohistochemical staining.

To monitor the levels of IL-37 expression in BALB/c mice, the mice were injected with pcDNA3.1-IL-37 plasmids using hydrodynamic tail vein injection [20,21] and sacrificed at 0, 6, 12, 24, 48, 72, and 96 h. Livers were collected for mRNA and protein extraction. We first checked IL-37 mRNA expression in the liver by RT-PCR. It was found that highest levels of IL-37 mRNA were present at 24 h after gene delivery. At the same time, IL-37 mRNA showed a time-dependent decrease (Figure 1(a)).

We then examine IL-37 protein levels in liver homogenate by ELISA to confirm the above results. As shown in Figure 1(b), it was found by ELISA that IL-37 protein was expressed in mouse liver homogenate after hydrodynamic tail vein injection of pcDNA3.1-IL-37 plasmid. Importantly, the levels of IL-37 protein in liver tissue showed similar trend as its mRNA levels described above (Figure 1(b)).

To further demonstrate the expression of IL-37 in mouse livers by hydrodynamic gene delivery of pcDNA3.1-IL-37 plasmid, we detected the expression of exogenous IL-37 protein in mouse livers by immunohistochemical staining. The results showed that we could detect the expression of IL-37 protein only after injection of pcDNA3.1-IL-37 plasmid (Figure 1(d)), while there was no expression of IL-37 after injection of control pcDNA3.1 plasmid (Figure 1(c))

### 3.2. IL-37 Protects Mice from Liver Injury Induced by APAP

**Figure 2:**
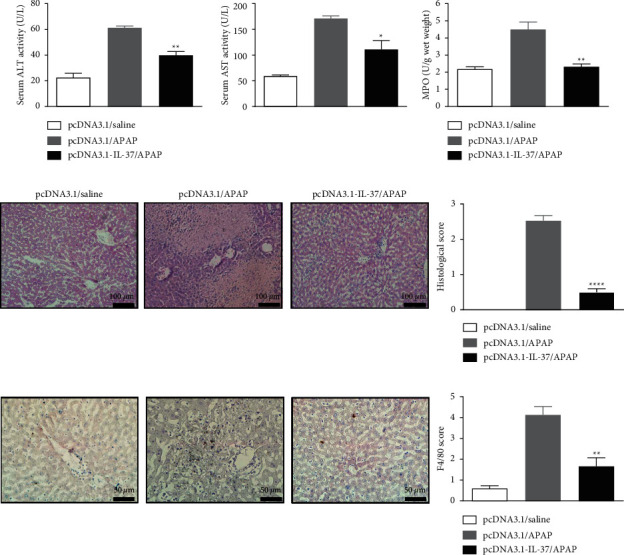
pcDNA3.1-IL-37 pretreatment attenuates APAP-induced liver injury. Mice underwent pretreatment with pcDNA3.1-IL-37 at 24 h before APAP challenge. The serum and liver tissue of animals were collected at 24 h after APAP injection. Liver sections obtained from animals at 24 h after APAP injection were subjected to histological analysis of hepatic necrosis. (a) Serum ALT and AST levels measured in each group. (b) Liver MPO activity. (c) Representative images of H&E staining chosen from each group. Bar graph shows the histopathological scores of inflammatory cells infiltration and liver necrosis. (d) Effect of IL-37 on the liver infiltrating Kupffer cells. Liver tissues were immunostained with anti-F4/80 antibody. Typical images were chosen from each group. Bar graph shows the scores of F4/80^+^ Kupffer cells. Data are expressed as mean ± SD (*n* = 6–8 per group, ^*∗*^*p* < 0.05, ^*∗∗*^*p* < 0.01, and ^*∗∗∗∗*^*p* < 0.0001 vs. the pcDNA3.1/APAP group).

To explore whether IL-37 protein has a protective effect on APAP-induced hepatotoxicity, the experimental liver injury model on BALB/c mice was established. Mice were injected plasmid pcDNA3.1 and pcDNA3.1-IL-37 using hydrodynamic tail vein injection. After 24 h of hydrodynamic procedure, the mice were then intraperitoneally injected APAP (400 mg/kg bodyweight). The mice were sacrificed at 24 h for analysis of liver injury. Blood and liver tissue were obtained at 24 h after APAP injection. As shown in Figure 2(a), serum ALT and AST levels in the pcDNA3.1/APAP group were significantly increased compared with the pcDNA3.1/saline group. Importantly, serum ALT and AST levels in the pcDNA3.1-IL-37/APAP group were lower than those in the pcDNA3.1/APAP group (Figure 2(a)). Hepatic MPO levels were increased in the APAP (400 mg/kg) group than that in the saline group. However, pcDNA3.1-IL-37 injection significantly decreased hepatic MPO levels compared with the pcDNA3.1/APAP group (*p* < 0.01, Figure 2(b)). The result demonstrates that pcDNA3.1-IL-37 treatment reduces neutrophil accumulation and inflammation in the liver.

The protective effect of pcDNA3.1-IL-37 pretreatment was further confirmed by analysis of liver tissue sections after 24 h of APAP injection. Compared with the pcDNA3.1/saline group, the pcDNA3.1/APAP group showed a serious liver injury as manifested by the spotted necrosis at 24 h after APAP induction. However, pcDNA3.1-IL-37/APAP pretreatment showed significant less severity of liver necrosis (Figure 2(c)). This histopathologic liver damage correlated with the elevated serum levels of the liver enzymes.

Kupffer cells, the resident macrophages in the liver, play a critical role in immune-mediated liver injury [24]. Therefore, we investigated the accumulation of Kupffer cells in the liver by immunohistochemistry using anti-F4/80 stain. Liver tissue sections after 24 h of APAP injection were stained with anti-F4/80 for Kupffer cells. In the pcDNA3.1/APAP group, more infiltrating F4/80^+^ Kupffer cells were observed around hepatic and portal venules and sinusoids in each section in comparison with the pcDNA3.1/saline group. The increase of the infiltrating F4/80^+^ Kupffer cells population in the pcDNA3.1-IL-37/ConA group was inhibited in comparison with the pcDNA3.1/APAP group (Figure 2(d)). These results indicate that IL-37 expression attenuates liver injury induced by APAP injection.

### 3.3. Effect of IL-37 on Serum Cytokine Levels of APAP-Induced Liver Injury

**Figure 3:**
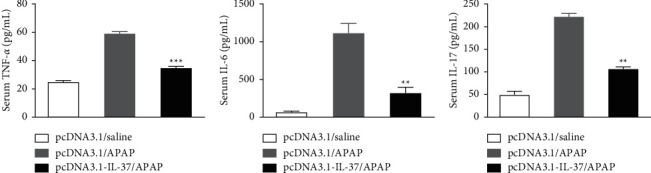
Effect of IL-37 expression on serum cytokine levels of APAP-induced liver injury. Mice were pretreated with pcDNA3.1-IL-37 at 24 h before APAP injection. Serum samples were collected at 24 h after APAP injection for the detection of proinflammatory and anti-inflammatory cytokines. Serum levels of TNF-*α* (a), IL-6 (b), and IL-17 (c) measured at 24 h after APAP injection by ELISA. Data are expressed as mean ± SD (*n* = 6–8 per group, ^*∗∗*^*p* < 0.01 1 and ^*∗∗∗∗*^*p* < 0.001 vs. the pcDNA3.1/APAP group).

APAP-induced liver injury is associated with the change of various inflammatory cytokines. To investigate whether IL-37 expression influences the systemic release of inflammatory cytokines, we first checked serum cytokine levels of TNF-*α*, IL-6, and IL-17 in mice after APAP treatment by ELISA. Proinflammatory cytokines TNF-*α*, IL-6, and IL-17 in serum from mice after APAP treatment were increased in pcDNA3.1/APAP in comparison with the pcDNA3.1/saline group. In contrast, it was noted that the increased serum cytokine levels of TNF-*α*, IL-6, and IL-17 were prevented in the pcDNA3.1-IL-37/APAP group (Figure 3).

### 3.4. Influence of IL-37 on the Expression of Inflammatory Cytokines mRNA and NF-*κ*B p65 Protein in the Liver Tissues

**Figure 4:**
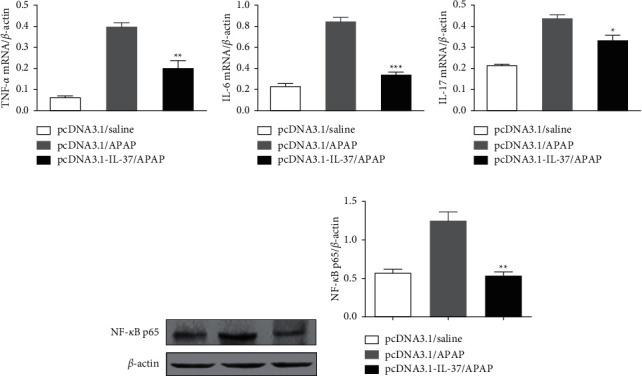
Influence of IL-37 on expression of inflammatory cytokines mRNAs and NF-*κ*B p65 proteins in APAP-induced liver injury. Mice were pretreated with pcDNA3.1-IL-37 at 24 h before APAP injection. Liver tissue samples were collected at 24 h after APAP administration for the detection of TNF-*α* (a), IL-6 (b), and IL-17 (c) by RT-PCR. Liver tissues were collected at 24 h after APAP administration for Western blot detections (d) and the corresponding densitometry analysis (e) of NF-*κ*B p65. *β*-Actin was used as an internal control. Data are expressed as mean ± SD (*n* = 6–8 per group, ^*∗*^*p* < 0.05, ^*∗∗∗*^*p* < 0.001 , and ^*∗∗∗*^*p* < 0.001 vs. the pcDNA3.1/APAP group).

To further confirm the above observations, we then checked mRNA levels of cytokines from the collected liver at 24 h after APAP treatment by RT-PCR. The results showed that the mRNA levels of proinflammatory cytokines TNF-*α*, IL-6, and IL-17 in the liver tissues of the pcDNA3.1-IL-37/APAP group were significantly decreased in comparison with the pcDNA3.1/APAP group. The data detected by RT-PCR were similar to those detected by ELISA (Figures 4(a)–4(c)). Western blot analysis showed that APAP can upregulate NF-*κ*B p65 protein expression in the liver tissues; however, IL-37 treatment significantly decreased APAP-induced NF-*κ*B p65 activation (Figures 4(d) and 4(e)). The results demonstrated that IL-37 inhibited inflammation by negatively regulating the NF-*κ*B signaling pathway.

## 4. Discussion

In the present study, we demonstrated that human IL-37 could be expressed in mouse liver by hydrodynamic tail vein injection of pcDNA3.1-IL-37 plasmid. IL-37 expression significantly reduced APAP-induced liver injury, serum ALT and AST levels, MPO activity, and inflammatory cells (neutrophils and macrophages) infiltration in the liver. Moreover, IL-37 expression significantly decreased proinflammatory cytokines (TNF-*α*, IL-6, and IL-17) levels in serums and liver tissues. These results suggest that IL-37 expression can protect mice from APAP-induced hepatotoxicity.

It has been reported that high levels of foreign gene expression in mouse hepatocytes can be achieved by the rapid injection of a large volume of naked plasmid DNA solution into the tail vein [25, 26]. Although a mouse homologue of IL-37 has not been reported, human IL-37 is functional in the mouse [8]. Therefore, we introduced a plasmid that expresses human IL-37 into mice using hydrodynamic tail vein injection. Indeed, the hydrodynamic tail vein procedure successfully delivered IL-37 into mouse liver by the detection of IL-37 mRNA and protein in liver tissue. Moreover, it was found that the highest levels of IL-37 mRNA and protein were presented at 24 h after gene delivery (Figure 1). Therefore, APAP was injected into mice at 24 h after gene delivery.

In this study, we demonstrated that IL-37 expression could inhibit APAP-induced liver inflammation and necrosis. Serum ALT and AST activity and liver histopathology are widely used as conventional indicators for the evaluation of liver injury. Our findings revealed that pcDNA3.1-IL-37 plasmids delivery significantly decreased hepatic injury, as judged by reduced serum ALT and AST activity, histopathological changes, and the ratio of necrotic to liver tissue in area (Figure 2).

The migration of neutrophils to mouse liver induced by APAP can be indirectly evaluated by measuring MPO activity [27, 28]. APAP-induced liver injury was accompanied by neutrophil infiltration, which was proved by the increase of MPO activity [29, 30]. Our results showed that pretreatment with pcDNA3.1-IL-37 plasmid significantly reduced APAP-induced MPO activity (Figure 2). Kupffer cells are the resident macrophages in the liver and play a critical role in the innate immune response and produce cytokines and chemokines under activated conditions. Activated Kupffer cells triggered by hepatocyte damage may lead to increased release of proinflammatory cytokines such as TNF-*α*, IL-6, and IL-17. So, we examined the number of Kupffer cell infiltration in the liver. F4/80 is a representative surface marker of mouse mononuclear phagocytes. It is a stable antigen and is not usually present in other types of leukocytes [31, 32]. Infiltrated Kupffer cells were defined by the expression of F4/80, and we found that IL-37 significantly inhibited the increase of F4/80^+^ Kupffer cells after APAP injection (Figure 2(d)).

Previous studies showed that the prognosis of patients with APAP-induced liver injury is related to inflammation [33, 34]. Excessive APAP can activate macrophages (Kupffer cells), recruit other inflammatory cells (such as lymphocytes and neutrophils), and lead to downstream inflammatory response [35]. Studies have found that APAP can upregulate NF-*κ*B p65 protein expression and inflammatory cytokines production of including IL-1*β*, IL-6, IL-17, and TNF-*α* in the liver tissues [29, 36, 37]. Roh et al. [38] demonstrated that overexpression of IL-17 by hydrodynamic tail vein injection of plasmid pcDNA3.1-IL-17 greatly enhanced the severity of liver injury. In this study, we observed a significant reduction in the TNF-*α*, IL-6, and IL-17 levels in serum as well as mRNA expression in the liver with treatment by IL-37 (Figures 3 and 4). Moreover, IL-37 treatment reduced NF-*κ*B p65 protein expression levels in the liver tissues of APAP-treated mice (Figure 4). Our findings suggest that the protective effect of IL-37 on APAP-induced liver injury is related to its inhibition of NF-*κ*B signal pathway activation and downregulation of subsequent proinflammatory cytokine expression.

## 5. Conclusion

To the best of our knowledge, this is the first report on the effect of IL-37 expression in the APAP-induced hepatotoxicity model in mice. The protective effect of IL-37 expression on APAP-induced liver injury is closely related to the inhibition of neutrophil migration and Kupffer cell activation, which results in the decrease of ALT, AST, and proinflammatory cytokines levels. These results indicate that IL-37 is a potential novel hepatoprotective agent.

## Figures and Tables

**Table 1:** Primer sequences used in this study.

Gene	Gene primers	Amplified fragment length (bp)
IL-37	Forward 5′-CGG GGT ACC ATG TCC TTT GTG GGG GAG-3′	657
	Reverse 5′-GCT CTA GAC TAA TCG CTG ACC TCA CTG-3′	

TNF-*α*	Forward 5′-TCT TCT CAT TCC TGC TTG TGG-3′	200
	Reverse 5′-CAC TTG GTG GTT TGC TAC GAC-3′	

IL-6	Forward 5′-GTG ACA ACC ACG GCC TTC CCT ACT-3′	313
	Reverse 5′-GGT AGC TAT GGT ACT CCA-3′	

IL-17	Forward 5′-TATCCCTCTGTGATCTGGGAAG-3′	161
	Reverse 5′-ATCTTCTCGACCCTGAAAGTGA-3′	

*β*-Actin	Forward 5′-TCC TGT GGC ATC CAT GAA ACT-3′	315
	Reverse 5′-GAA GCA CTT GCG GTG CAC GAT-3′	

## Data Availability

The datasets used and/or analyzed during the current study are available from the corresponding author upon request.
